# Exercise increases the release of NAMPT in extracellular vesicles and alters NAD ^+^ activity in recipient cells

**DOI:** 10.1111/acel.13647

**Published:** 2022-06-03

**Authors:** Mee Chee Chong, Anabel Silva, Patrick F. James, Sam Shi Xuan Wu, Jason Howitt

**Affiliations:** ^1^ School of Health Sciences Swinburne University of Technology Melbourne Victoria Australia; ^2^ Exopharm Ltd Melbourne Victoria Australia; ^3^ Iverson Health Innovation Institute Swinburne University of Technology Melbourne Victoria Australia

**Keywords:** aging, exercise, exosome, extracellular vesicles, healthspan, NAD^+^, NAMPT, SIRT1

## Abstract

Aging is associated with a loss of metabolic homeostasis, with cofactors such as nicotinamide adenine dinucleotide (NAD^+^) declining over time. The decrease in NAD^+^ production has been linked to the age‐related loss of circulating extracellular nicotinamide phosphoribosyltransferase (eNAMPT), the rate‐limiting enzyme in the NAD^+^ biosynthetic pathway. eNAMPT is found almost exclusively in extracellular vesicles (EVs), providing a mechanism for the distribution of the enzyme in different tissues. Currently, the physiological cause for the release of eNAMPT is unknown, and how it may be affected by age and physical exercise. Here, we show that release of small EVs into the bloodstream is stimulated following moderate intensity exercise in humans. Exercise also increased the eNAMPT content in EVs, most prominently in young individuals with higher aerobic fitness. Both mature fit and young unfit individuals exhibited a limited increase in EV‐eNAMPT release following exercise, indicating that this mechanism is related to both the age and physical fitness of a person. Notably, unfit mature individuals were unable to increase the release of eNAMPT in EVs after exercise, suggesting that lower fitness levels and aging attenuate this important signalling mechanism in the body. EVs isolated from exercising humans containing eNAMPT were able to alter the abundance of NAD^+^ and SIRT1 activity in recipient cells compared to pre‐exercise EVs, indicating a pathway for inter‐tissue signalling promoted through exercise. Our results suggest a mechanism to limit age‐related NAD^+^ decline, through the systemic delivery of eNAMPT via EVs released during exercise.

## Graphical abstract

Aging is associated with decreased NAD^+^ production, which is critical for maintaining tissue and metabolic homeostasis. Here, we identified that moderate intensity exercise in humans can result in the release of NAMPT in extracellular vesicles, that when delivered to recipient cells promotes NAD^+^ biosynthesis and SIRT1 activity. Our findings show an exercise‐based mechanism for the production of NAD^+^ that differs between age groups, moderated by the release of eNAMPT in EVs.

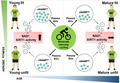

## 1 INTRODUCTION, RESULTS AND DISCUSSION

Nicotinamide adenine dinucleotide (NAD^+^) is an essential metabolite for energy metabolism that is involved in key cellular processes including glycolysis, the Krebs cycle, DNA repair, and other metabolic reactions within the cell (Canto et al., 2015; McReynolds et al., 2020). NAD^+^ levels have been identified to decline during aging, with alterations in NAD^+^ homeostasis being found in virtually all age‐related diseases, including neurodegeneration, diabetes, and cancer. Nicotinamide phosphoribosyltransferase (NAMPT) is a rate‐limiting enzyme in the NAD^+^ biosynthetic pathway that exists in intra‐ and extra‐cellular forms (known as iNAMPT and eNAMPT, respectively; Yoshino et al., 2018). Recently, it has been reported that there is a decline in circulating eNAMPT levels in older humans and rodents, resulting in the age‐related loss of systemic NAD^+^ levels (Yoshida et al., 2019).

eNAMPT has been found to reside almost exclusively in extracellular vesicles (EVs), which are thought to provide an inter‐tissue communication mechanism, delivering eNAMPT to cells and enhancing NAD^+^ activity (Yoshida et al., 2019). In rodent studies, delivery of EVs containing eNAMPT derived from young mice were able to enhance physical activity, lifespan, and healthspan in aged mice, suggesting that the loss of eNAMPT over time could be responsible for age‐related physiological decline (Yoshida et al., 2019). However, the role of aging, and the contribution of physical activity to the release of eNAMPT in humans is currently unknown. Here, we investigated the release of eNAMPT in EVs after moderate intensity exercise, in both young and mature individuals, to determine the influence that aging and exercise has on extracellular signalling pathways involved in NAD^+^ biosynthesis.

**FIGURE 1:**
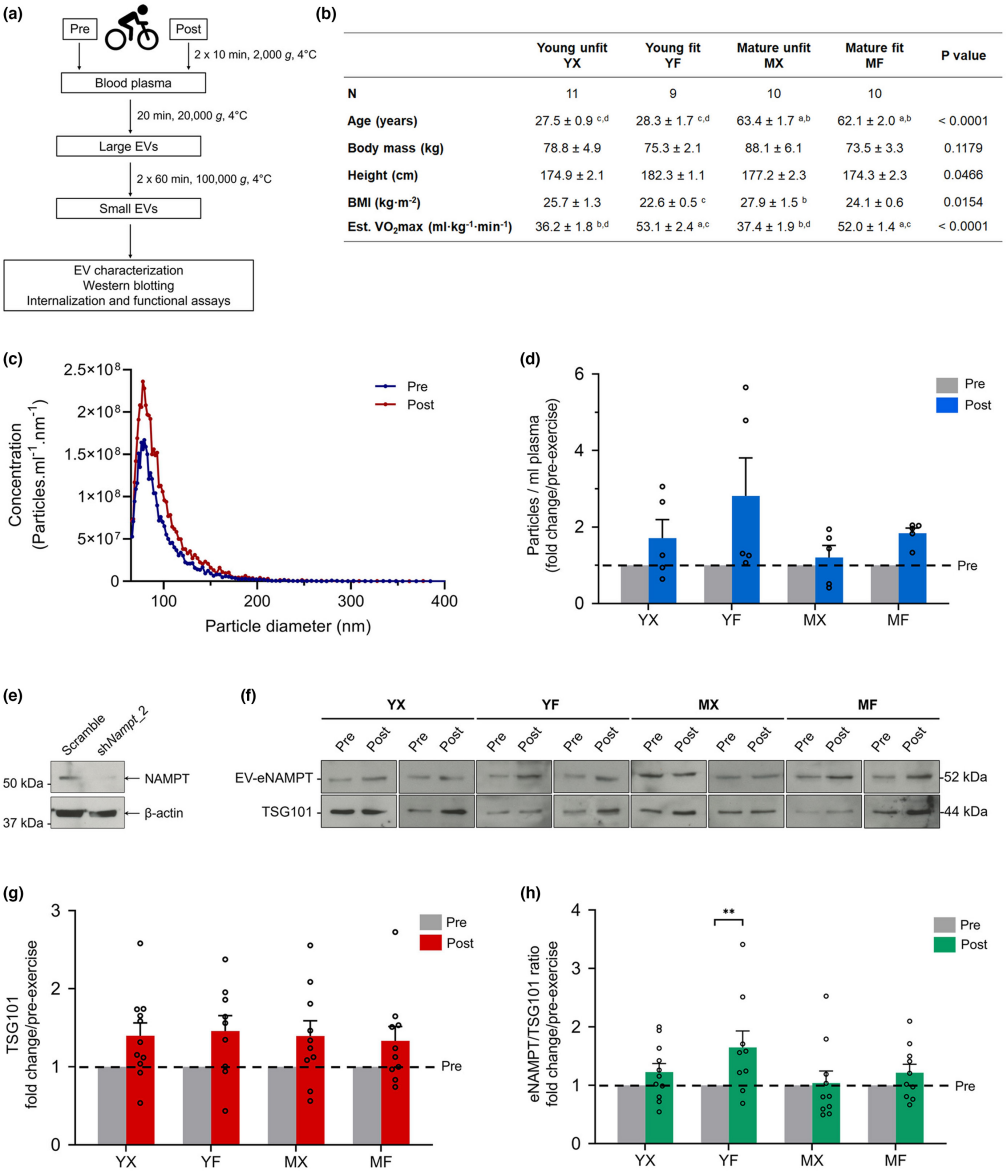
Characterization of EVs released following exercise in healthy adults of different ages and fitness levels. (a) a schematic diagram of the experimental design. (b) the characteristics of the study participants (*n* = 9–11 per group). Est. VO_2_max, estimated maximum oxygen consumption. (a), (b), (c), and (d) denote the columns and the statistical differences. (c) Representative size distribution profile of EVs of a participant, here showing an MX group participant collected at pre‐ and post‐exercise. (d) Individual (circle) and mean (bar) fold changes of total particle counts of EVs collected at pre‐ and post‐exercise (*n* = 5 per group). (e) Western blot for NAMPT in C2C12 cell lysates with either scrambled or shRNA knockdown of NAMPT. (f) Representative Western blot analysis of TSG101 and eNAMPT from exercise‐EVs. (g) Individual (circle) and mean (bar) fold changes of densitometric quantification of TSG101 signal (*n* = 9–11 per group). (h) Individual (circle) and mean (bar) fold changes of densitometric quantification of EV‐eNAMPT signal normalized to TSG101 (*n* = 9–11 per group). Data in (b), (d), (g), and (h) represents the mean ± SEM, *p < 0.05, **p < 0.01. Post‐exercise expressed in relation to pre‐exercise (dashed line)

To investigate the effect of exercise on eNAMPT in humans, 40 healthy males were subjected to a 20 min cycling exercise at 70% of estimated VO_2max_. Participants were classified into four groups (young unfit, YX; young fit, YF; mature unfit, MX; mature fit, MF) based on age (young, 18–35 years; mature, 50–70 years) and aerobic fitness (unfit, estimated VO_2max_ < 45 ml^.^kg^−1^ min^−1^; fit, estimated VO_2max_ > 45 ml^.^kg^−1^ min^−1^) (Figure 1a,b). Blood plasma was collected both pre‐ and post‐exercise, and EVs were isolated using differential ultracentrifugation. Following isolation, we characterized exercise EVs using a Spectradyne nCS1 with a TS‐400 cartridge. The isolated exercise EVs exhibited a mean size of 88 ± 4.7 and 89 ± 4.9 nm (± SD) at pre‐ and post‐exercise, respectively, indicating that particle size did not change with exercise, and was consistent across age and fitness levels (Figure 1c). We observed that for each group, the amount of EVs at baseline (pre‐exercise) was similar, with no significant difference observed between the four groups (Figure S1). Mixed effects model analysis of particle counts revealed a significant increase in the overall release of EVs into the bloodstream from pre‐ to post‐exercise (*p* < 0.01, Figure 1d). However, given the limited number of samples analysed, we did not test for significant differences in any individual participant group.

To test the validity of the NAMPT antibody used in this study, we knocked down (KD) *Nampt* in C2C12 myoblasts using a shRNA. Lysates from scramble control and *Nampt* KD C2C12 cells were subjected to Western blotting and probed with an antibody toward NAMPT (Adipogen, OMNI379). The Western blot signal detected in scramble control lane ran at the expected molecular mass of 52 kDa (Figure 1e). We observed that *Nampt* KD decreased NAMPT protein abundance (normalized to β‐actin) compared with scramble control, confirming the specificity of the NAMPT antibody. To identify the release of eNAMPT in EVs after exercise, we performed Western blots with antibodies toward both NAMPT and the small EV marker TSG101. There was a significant main effect from pre‐ to post‐exercise for TSG101 abundance (*p* < 0.001), indicating a significant increase in the release of a subset of small EVs into the circulation post exercise independent of group (Figure 1f,g). However, there was no significant main effect of individual groups for TSG101 abundance (*p* > 0.05). Next, we investigated the amount of eNAMPT in EVs released after exercise in each group. We observed that both young groups, and the mature fit group had an increase in eNAMPT after exercise, however, the mature unfit group showed no increase post‐exercise (Figure 1f,h). Post‐hoc analysis showed that the young fit participants were the only group that had a significant increase in eNAMPT in EVs after exercise (*p* < 0.01, Figure 1f,h).

**FIGURE 2:**
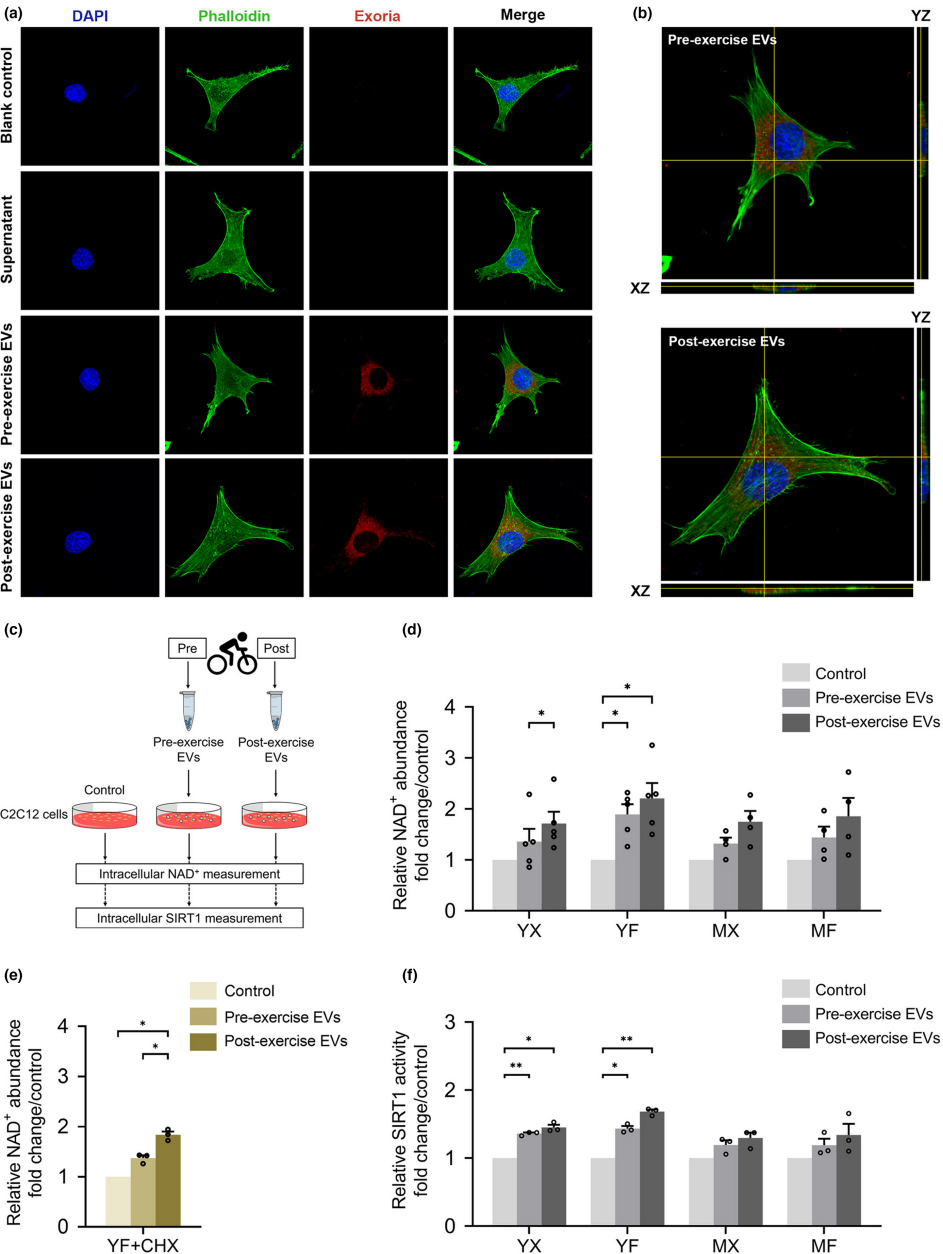
Pre‐ and post‐exercise EVs are internalized into recipient cells and increase NAD^+^ and SIRT1 activity. (a) Representative images of Exoria™ labelled exercise EVs distribution after incubation with C2C12 myoblasts that are labeled with FITC phalloidin and DAPI nuclear stain. Pre‐ and post‐exercise EV samples of a participant from the MF group observed here. (b) Orthogonal view of C2C12 myoblasts confirmed internalization of the EVs across the cell membrane. (c) An overview of the exercise EV uptake assay to examine the role of EV‐eNAMPT in NAD^+^ biosynthesis and SIRT1 activity. (d) Relative NAD^+^ abundance in C2C12 myoblasts after treatment with EVs isolated from pre‐ and post‐exercise plasma for each group (*n* = 4–5 per condition). (e) Relative NAD^+^ abundance in C2C12 myoblasts after treatment with EVs isolated from pre‐ and post‐exercise plasma of the YF group with cycloheximide treatment (CHX) (*n* = 3 per condition). (f) Relative SIRT1 activity in C2C12 myoblasts after treatment with EVs isolated from pre‐ and post‐exercise plasma (*n* = 3 per condition). Data in (d), (e), and (f) represents mean ± SEM, *p < 0.05, **p < 0.01

To assess if EVs derived from exercising humans containing eNAMPT could alter NAD^+^ abundance in recipient cells, we performed cellular uptake experiments combined with NAD^+^ assays. To measure cellular uptake, pre‐ and post‐exercise EVs were labelled using the fluorescent dye Exoria™ (Tertel et al., 2022) and added to the media of C2C12 myoblasts for 60 min before the cells were fixed and mounted. Confocal microscopy revealed the presence of a red fluorescent signal in C2C12 myoblasts treated with either pre‐ or post‐exercise EVs, suggesting the internalization of the EVs (Figure 2a). Z‐stack imaging of the cells confirmed internalization of the EVs across the cell membrane (Figure 2b). To exclude the possibility of free dye uptake to the cells, supernatant derived from the staining procedure containing Exoria™ dye was used as a control, no red fluorescent signal was observed in these samples (Figure 2a, row 2). To identify the effect of exercise EVs on cellular NAD^+^ biosynthesis, we delivered pre‐ and post‐exercise EVs to C2C12 myoblasts and measured the NAD^+^ abundance in the recipient cells using a commercially available colorimetric assay (Figure 2c). We observed significant increases in NAD^+^ abundance after exercise EVs from the YX and YF groups were delivered to the recipient cells (*p* < 0.05; Figure 2d). The increase in NAD^+^ was observed 60 min after delivery of EVs, suggesting a direct effect of eNAMPT delivery. However, to test if delivery of exercise‐EVs were able to upregulate protein expression that resulted in changes to NAD^+^, we pre‐treated C2C12 myoblasts with cycloheximide (CHX) to inhibit de novo protein translation. In the presence of CHX, the significant increase in the NAD^+^ abundance was maintained following the delivery of post‐exercise EVs, compared with control and pre‐exercise EVs from the YF group (*p* < 0.05; Figure 2e). Indicating that exercise EVs could directly alter metabolic pathways in recipient cells without changes in protein expression. It should be noted that we cannot fully rule out another mechanism that may be activated after delivery of EVs to increase NAD^+^. However, given the timeframe of our experiments and controls, we believe that our results support the delivery of EV‐eNAMPT as the main contributor to the observed changes.

Next, we tested the impact of exercise EVs on Sirtuin 1 (SIRT1), an NAD^+^‐dependent enzyme, by measuring the SIRT1 activity in recipient cells using a commercially available fluorometric assay (Figure 2c). Both pre‐ and post‐exercise EVs from YX and YF groups significantly upregulated SIRT1 activity in the recipient cells compared with control (*p* < 0.05 and *p* < 0.01; Figure 2f). While statistical significance was not achieved, we observed small increases in SIRT1 activity after exercise EVs from the MX and MF groups were delivered. These results suggest that pathways downstream of eNAMPT can be activated in recipient cells after delivery of EVs.

Exercise remains the most promising intervention to improve health and physical function in older adults, however, at a cellular level, our understanding for the multisystem effects of exercise are not well understood. The cell intrinsic effects of iNAMPT in skeletal muscle have been shown to increase after exercise, with the ability to regulate skeletal muscle function during aging (Costford et al., 2010; de Guia et al., 2019; Koltai et al., 2010; Lamb et al., 2020). However, the cell extrinsic role of eNAMPT has not been investigated in the context of exercise and aging. Here, we find that exercise increases the release of EVs, as well as the eNAMPT content in EVs (EV‐eNAMPT), predominantly in young individuals with higher aerobic fitness, indicating that this mechanism is related to both age and physical activity level of an individual. We found that mature individuals with lower aerobic fitness did not show any increased release of EV‐eNAMPT in response to exercise. It is unclear if this is because of lower NAMPT availability in this age group, or if the EV packaging mechanism is attenuated in older unfit individuals. It is interesting to note that the mature fit group displayed similar amount of EV‐eNAMPT release after exercise to that of the young unfit group, suggesting that aerobic fitness may overcome some of the age‐related decline in eNAMPT release. The exact mechanism for how eNAMPT is secreted in EVs during exercise remains unclear; however, it has been demonstrated that eNAMPT is secreted from adipose tissue through SIRT1‐dependent deacetylation of iNAMPT (Yoon et al., 2015). It will be important to identify how exercise can influence this mechanism, or other pathways that can release eNAMPT in EVs.

Our results suggest that release of EV‐eNAMPT is able to influence the abundance of NAD^+^ and SIRT1 activity in recipient cells, indicating a mechanism where exercise can result in cross tissue signalling to maintain metabolic homeostasis in multiple organs. However, whilst we observed significant changes with EVs from younger individuals, this was not observed in EVs from mature participants. This is of particular value, given that age‐related decline in circulating eNAMPT has been shown to limit NAD^+^ production (Yoshida et al., 2019). Importantly, the availability of NAD^+^ in a cell regulates the activity of the sirtuin family of NAD^+^‐dependent deacetylases (which conversely can regulate eNAMPT production). Multiple animal studies have implicated the dysregulation of sirtuins and NAD^+^ in aging and age‐related metabolic disruption (Balan et al., 2008; Mouchiroud et al., 2013; Rogina & Helfand, 2004; Tissenbaum & Guarente, 2001). However, the ability to extend lifespan with NAD^+^ supplementation or sirtuin overexpression has not been consistent, with evidence suggesting tissue specific roles for this pathway in aging (Banerjee et al., 2012; Hoffmann et al., 2013; Satoh et al., 2013). Significantly, EVs have been shown to communicate to specific cells and tissues in vivo (Ridder et al., 2014; Schnatz et al., 2021), highlighting their ability to function in biologically effective pathways with the capacity to deliver eNAMPT to specific tissues. Currently, we are limited in our understanding of the donor cells responsible for the EV‐eNAMPT detected in the bloodstream, as well as the recipient cells that internalize these EVs after exercise. Surface marker characterization of EVs is an emerging field that in the future will enable identification of EV‐eNAMPT release and uptake in vivo.

In this study, we used moderate intensity exercise (as recommended by the American College of Sports Medicine, 2013) to investigate EV release mechanisms in the body. This is in contrast to previous studies that have employed trained athletes exercising until exhaustion (Brahmer et al., 2019; Fruhbeis et al., 2015). As such, our findings are better able to inform on EV release in response to habitual daily exercise settings, particularly in mature adults. Together, our results suggest a mechanism for the systemic delivery of eNAMPT via EVs released during exercise, to maintain and counteract the decline in tissue NAD^+^ levels. Our findings illustrate the important role that exercise has in promoting cellular crosstalk via EVs, to improve health during aging.

## 2 AUTHOR CONTRIBUTIONS

J.H. and M.C.C. conceived the project. J.H., S.W. and M.C.C. designed the experiments. M.C.C. performed data collection. M.C.C., A.S., and P.F.J. performed and analyzed the experiments. M.C.C. and J.H. analyzed the data, prepared the figures, and wrote the manuscript. All authors commented and approved the manuscript.

## 4 CONFLICT OF INTEREST

A.S. and P.F.J. are employees of Exopharm Ltd. P.F.J. is a shareholder of Exopharm. J.H. is a scientific consultant to Exopharm Ltd.

5

## Supporting information


Figure S1

Appendix S1

## Data Availability

Data are available upon request from the corresponding author.
