# Supplementary material for: Exercise increases the release of NAMPT in extracellular vesicles and alters NAD + activity in recipient cells
Source: Aging Cell. 2022 Jun 3;21(7):e13647. doi: 10.1111/acel.13647 (PMC9282849; doi:10.1111/acel.13647)

**Figure S1** Total particle counts of EVs pre-exercise in healthy adults of different ages and fitness levels. Individual (circle) and mean (bar) fold changes of total particle counts of EVs collected at pre-exercise (n = 5 per group). One-way ANOVA showed no significant difference between the groups. Data represents the mean ± SEM.


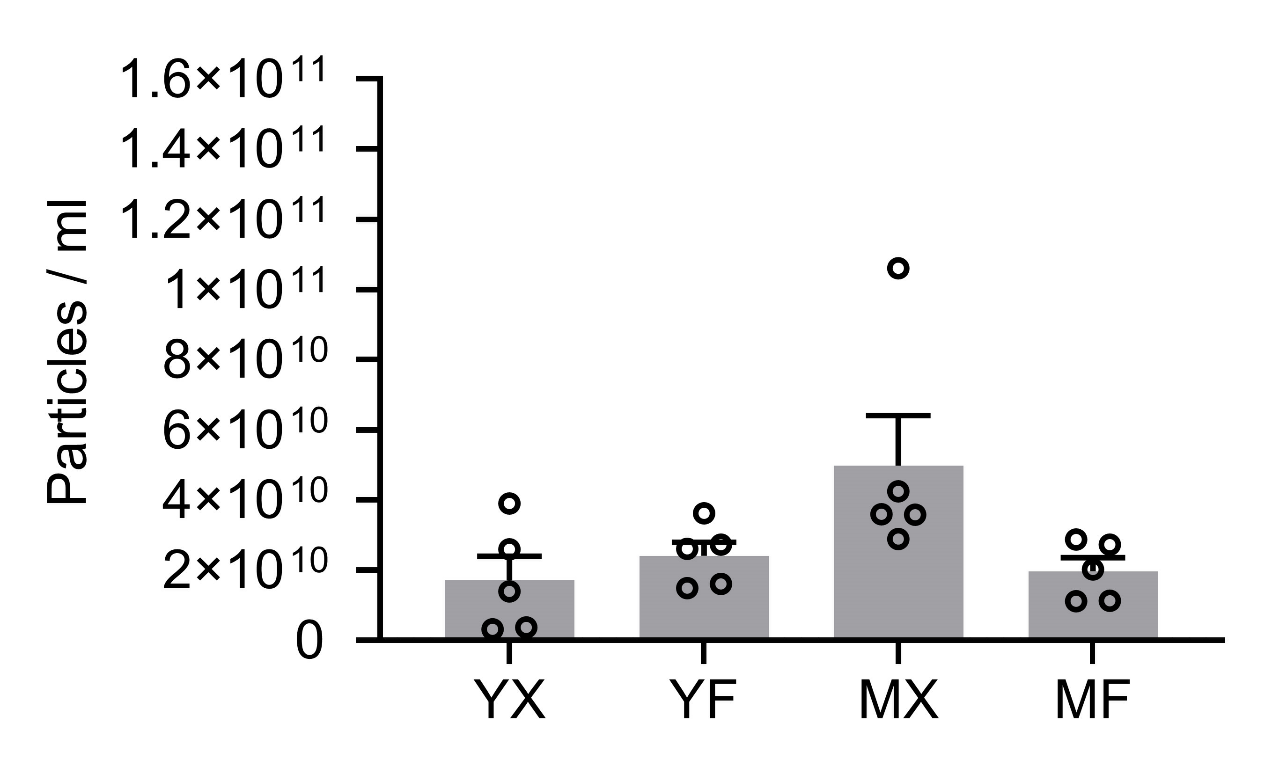

Supplement: Supplementary file 1 — Figure S1 [file ACEL-21-e13647-s002.docx]
