# Supplementary material for: Exercise increases the release of NAMPT in extracellular vesicles and alters NAD + activity in recipient cells
Source: Aging Cell. 2022 Jun 3;21(7):e13647. doi: 10.1111/acel.13647 (PMC9282849; doi:10.1111/acel.13647)
Supplement: Supplementary file 2 — Appendix S1 [file ACEL-21-e13647-s001.docx]

**Experimental procedures**

**Ethical approval**

All experimental procedures were approved by Swinburne Human Research Ethics Committee in accordance with the standards of the Declaration of Helsinki of the World Medical Association. All participants were informed of the procedures and possible risks associated with the experiments, and provided written consent to participate in the study.

**Participants and exercise design**

Forty healthy males voluntarily participated in this study. The inclusion criteria were aged between 18 to 35 or 50 to 70 years, healthy with no known clinical disease, and free from lower body injury 2 months prior to participation. Participants were asked to attend two experimental trials separated by at least 48 h. Prior to both experimental trials, participants were told to abstain from strenuous physical activity for 24 h, caffeine for 8 h, and food for 2 h.

During the first experimental trial, participants were asked to perform a YMCA submaximal cycle ergometer test to estimate their maximum oxygen consumption (VO_2max_). The YMCA submaximal cycle ergometer test was conducted as described by Golding, Myers, and Sinning (1982). In brief, participant began exercising on a cycle ergometer at 25 W at a cadence of 50 rpm. Power output progression was based on the participant’s HR response to the first stage of exercise (if HR < 80 beats·min^-1^: 125 W; 80 to 90 beats·min^-1^: 100 W; 90 to 100 beats·min^-1^: 75 W; and > 100 beats·min^-1^: 50 W), and each stage of the test lasted 3 min. If the participant’s HR varied > 5 beats·min^-1^ between the last 2 min of each stage, an additional minute was added to that stage. This method was used until HR for the last 2 min were within ± 5 beats·min^-1^. The test was ceased when two workloads were completed with HR between 110 and < 85% of their age-predicted maximum. An extra stage or more were added if the participant’s HR did not exceed 110 beats·min^-1^ during the previous stages. Those extra stages were only necessary for very fit participants (Garatachea, Cavalcanti, Garcia-Lopez, Gonzalez-Gallego, & de Paz, 2007). VO_2max_ was estimated according to the ACSM’s guidelines (American College of Sports Medicine, 2013).

During the second experimental trial, participants were asked to perform a 20 min continuous cycling exercise at a power output that elicited approximately 70% of individual estimated VO_2max_ determined from the YMCA submaximal cycle test. Before and immediately after the cycling exercise, 34 ml of venous blood samples were withdrawn from the medial cubital vein by venepuncture using a 21-gauge BD Vacutainer^®^ Safety-Lok™ needle (BD Biosciences, Oxford, UK) into Acid Citrate Dextrose Vacutainer^®^ (ACD-A, BD Biosciences, Oxford, UK). Blood samples were kept vertically on ice during transport to the laboratory.

**Venous plasma preparation and EV isolation**

Blood was centrifuged at 2,000 x *g* for 10 min at 4°C. Plasma was collected and subjected to a second centrifugation at 2,000 x *g* for 10 min at 4°C to obtain platelet-free plasma. Platelet-free plasma was always kept on ice until EV isolation. Isolation of EVs was achieved by a three-step differential centrifugation protocol with two ultracentrifugation steps. Differential ultracentrifugation has been shown to isolate a greater purity and higher functionality of small EVs from blood plasma samples that are appropriate for downstream applications such as functional studies (An, Wu, Zhu, & Lubman, 2018; Takov, Yellon, & Davidson, 2019). Platelet-free plasma was transferred to a Beckman Thickwall Polypropylene centrifuge tube (Beckman Coulter, Krefeld, Germany) and centrifuged at 20,000 x *g* for 20 min at 4°C in a fixed angle rotor (Type 70Ti, Beckman Coulter, Krefeld, Germany). The supernatant was then transferred to clean centrifuge tubes, diluted with ice-cold phosphate-buffered saline (PBS) and centrifuged at 100,000 x *g* for 60 min at 4°C. The supernatant was removed, the remaining pellet was washed in ice-cold PBS and centrifuged again at 100,000 x *g* for 60 min at 4°C. The supernatant was carefully removed, and the small EV pellet (resulting from 100,000 x *g* centrifugation) was resuspended by repeated pipetting. For Western blot (WB) analysis, EV pellet was resuspended in Laemmli buffer and heated at 95°C for 5 min. For other analyses, EV pellet was resuspended in PBS. All samples were stored at -80°C until further analysis.

**EV characterization**

EV size and particle counts were conducted using the nCS1 instrument (Spectradyne, Torrance, CA, USA). Pre- and post-exercise EV samples were diluted to 1:10 in 20 nm filtered PBS containing 0.2% polysorbate. Five µl of diluted samples were loaded onto TS-400 cartridges, allowing measurement for particles between 65 to 400 nm. Particle acquisition was carried out with the loaded cartridges using the Spectradyne nCS1 instrument and software. All acquired results were analysed using the Data Viewer software. Final particle counts were calculated by determining the diameter factor using standard sized beads and the concentration factor, and subtracting the blank.

**Western blot analysis**

EV pellets resuspended in Laemmli buffer were subjected to sodium dodecyl sulfate-polyacrylamide gel electrophoresis (SDS-PAGE) and Western blotting. Samples were separated by SDS-PAGE, and transferred to polyvinylidene difluoride membrane (PVDF, Bio-Rad, Hercules, CA, US). Membranes were blocked with 5% milk powder in Tris-buffered saline containing 0.05% Tween-20 (TBST) and incubated sequentially with primary antibodies for overnight at 4°C and HRP-coupled secondary antibodies for 60 min at room temperature. Proteins were detected using an enhanced chemiluminescence (ECL) reagents (GE Healthcare, Chicago, IL, USA) on X-ray films. ImageJ software (version 1.52a, National Institutes of Health (NIH), Bethesda, MD, USA) was used for the semi-quantitative analysis of WB signals. Commercially available antibodies were used including rabbit anti-Tsg101 (Sigma-Aldrich, 1:1,000) and mouse anti-Nampt (Adipogen, 1:1,000). Secondary antibodies that were used are horseradish peroxidase (HRP)-conjugated goat anti-rabbit antibody and HRP-conjugated goat anti-mouse antibody (Upstate Biotech, 1:5,000).

**Cell culture**

C2C12 mouse skeletal muscle cells (myoblasts) were grown in Dulbecco’s modified Eagle’s medium (DMEM) supplemented with 10% foetal bovine serum (FBS), 1% _L_-glutamine and 0.02% penicillin-streptomycin at 37°C humidified with 5% CO_2_. Experiments were performed with cells in passages 5-12 and within 48 h post-seeding when cell confluence was 80-90%, unless otherwise stated. For experiments with protein translation inhibitor, cells were treated in media containing 10 µg/ml cycloheximide (CHX; Sigma) for 6 hours at 37°C prior to experimentation.

*Nampt* knockdown (KD) in C2C12 myoblasts was generated using short hairpin (sh)RNA delivered by plasmid transfection. Two separate shRNA sequences (sh*Nampt_*1: RMM3981-201818874 and sh*Nampt_*2: RMM3981-201824136; Open Biosystems, AL, USA) and a scramble control (pLKO.1 empty vector control; Open Biosystems, AL, USA) were used, as described previously (Agerholm et al., 2018; Brandauer et al., 2013). Cells were transfected using Effectene transfection reagent (Qiagen) according to manufacturer’s instructions. At 24 h after transfection, cells were maintained in selection media containing DMEM supplemented with 10% FBS, 1% _L_-glutamine, 0.02% penicillin-streptomycin and 1 µg/ml puromycin. We observed higher knockdown efficiency using the sh*Nampt_*2 construct.

**EV internalization assay**

C2C12 cells (2.5 x 10^4^) were cultured on poly-d-lysine (PDL)-coated glass coverslips for 24 h. EVs were labelled with a fluorescent dye Exoria^TM^ (Exopharm, Melbourne, Australia) (Tertel et al., 2022) prior to incubation on C2C12 cells. The working solution of Exoria^TM^ was 2 µM in PBS, obtained by a dilution of a 2 mM solution in ethanol. For EV labelling, 25 µl of EVs in PBS, corresponding to ~3 x 10^8^ EVs/ml of pre- and post-exercise samples respectively, was pipetted into equal amount of 2 µM Exoria^TM^ solution and incubated for 30 min at 37°C without exposure to the light. To remove unbound free dye, labelled EVs were washed twice in PBS at 100,000 x *g* for 60 min at 4°C and resuspended in HEPES buffer.

Exoria^TM^-labeled EVs were added to C2C12 cells grown on glass coverslips and incubated for 60 min at 37°C. Cells were washed with PBS and fixed in 4% PFA for 20 min at room temperature. Actin was visualized with fluorescein isothiocyanate (FITC)-labeled phalloidin and nuclei was counterstained with DAPI. Coverslips were mounted on glass slides and visualized using a confocal microscope. Z-stack images were acquired using an FV3000 confocal microscope (Olympus, Tokyo, Japan) equipped with an UPLSAPO 60x oil objective lens. Olympus FV31S-SW imaging software was used to acquire images and laser power in each channel was maintained constant for all samples. Images were exported for further analysis in Fiji ImageJ software (version 1.53c, NIH, Bethesda, MD, USA).

**NAD^+^ assay**

C2C12 cells were seeded at 3 x 10^5^/well in 12-well plates. For experiments in the presence of CHX, C2C12 cells were treated with media containing 10 µg/ml CHX for 6 hours at 37°C. Cells in the absence and presence of CHX treatment were then serum starved for 3 h in DMEM without FBS supplemented with 1% _L_-glutamine and 0.02% penicillin-streptomycin. Cells were incubated with or without 150 µg of EVs isolated from pre- and post-exercise plasma for 1 hour at 37°C. Intracellular NAD^+^ abundance was measured using the EnzyChrom NAD^+^/NADH Assay Kit (E2ND-100; BioAssay Systems) according to the manufacturer’s instructions. Briefly, cells were homogenized in 100 µl of NAD^+^ extraction buffer for NAD^+^ determination. Samples were heated at 60°C for 5 min, and then mixed with 20 µl of assay buffer and 100 µl of the opposite NADH extraction buffer to neutralize the extracts. Next, samples were briefly vortexed and centrifuged at 14,000 rpm for 5 min. NAD^+^ abundance in the supernatant was quantified by measuring the absorbance change at 560 nm using a microplate reader.

**SIRT1 assay**

C2C12 cells were seeded at 3 x 10^5^/well in 12-well plates and 24 h later serum starved for 3 h in DMEM without FBS supplemented with 1% _L_-glutamine and 0.02% penicillin-streptomycin. Cells were incubated with or without 150 µg of EVs isolated from pre- and post-exercise plasma for 2 hours at 37°C. Intracellular SIRT1 activity was measured using the SIRT1 Activity Assay Kit (ab156065; Abcam) according to the manufacturer’s instructions. Briefly, cells were harvested and lysed in lysis buffer. Samples were centrifuged at 14,000 rpm for 10 min at 4°C. Reactions were initiated, and fluorescence intensity was measured for 30-60 min at 1-2 min intervals on a microplate fluorometer with excitation at 340-360 nm and emission at 440-460 nm. SIRT1 activity was calculated with the difference in fluorescence intensity between samples and blank control when the SIRT1 reaction rate is maintained.

**Statistical analysis**

Data are presented as mean ± SEM, unless otherwise stated. All statistical analyses were performed using GraphPad Prism 9. Analyses of EV particle counts, TSG101, eNAMPT, NAD^+^ abundance, and SIRT1 activity were performed using linear mixed-effects models. Fixed and random effects for the linear mixed models were fit for each dependent variable and the main effects for group (YX, YF, MX, or MF) and time (pre- and post-exercise), as well as interactions (group x time). A Bonferroni post hoc pairwise comparison was performed if a significant main effect and/or interaction effect was present. A 95% confidence interval was used to define the level of significance. *p < 0.05, **p < 0.01. Sample sizes and other statistical parameters are indicated in the figure legends.

**References**

Agerholm, M., Dall, M., Jensen, B. A. H., Prats, C., Madsen, S., Basse, A. L., . . . Treebak, J. T. (2018). Perturbations of NAD(+) salvage systems impact mitochondrial function and energy homeostasis in mouse myoblasts and intact skeletal muscle. *Am J Physiol Endocrinol Metab, 314*(4), E377-E395. doi:10.1152/ajpendo.00213.2017

American College of Sports Medicine. (2013). *ACSM's guidelines for exercise testing and prescription*: Lippincott Williams & Wilkins.

An, M., Wu, J., Zhu, J., & Lubman, D. M. (2018). Comparison of an Optimized Ultracentrifugation Method versus Size-Exclusion Chromatography for Isolation of Exosomes from Human Serum. *J Proteome Res, 17*(10), 3599-3605. doi:10.1021/acs.jproteome.8b00479

Brandauer, J., Vienberg, S. G., Andersen, M. A., Ringholm, S., Risis, S., Larsen, P. S., . . . Treebak, J. T. (2013). AMP-activated protein kinase regulates nicotinamide phosphoribosyl transferase expression in skeletal muscle. *J Physiol, 591*(20), 5207-5220. doi:10.1113/jphysiol.2013.259515

Garatachea, N., Cavalcanti, E., Garcia-Lopez, D., Gonzalez-Gallego, J., & de Paz, J. A. (2007). Estimation of energy expenditure in healthy adults from the YMCA submaximal cycle ergometer test. *Eval Health Prof, 30*(2), 138-149. doi:10.1177/0163278707300628

Golding, L. A., Myers, C. R., & Sinning, W. E. (1982). *Y's Way to Physical Fitness*: Human Kinetics Publishers.

Takov, K., Yellon, D. M., & Davidson, S. M. (2019). Comparison of small extracellular vesicles isolated from plasma by ultracentrifugation or size-exclusion chromatography: yield, purity and functional potential. *J Extracell Vesicles, 8*(1), 1560809. doi:10.1080/20013078.2018.1560809

Tertel, T., Schoppet, M., Stambouli, O., Al-Jipouri, A., James, P. F., & Giebel, B. (2022). Imaging flow cytometry challenges the usefulness of classically used extracellular vesicle labeling dyes and qualifies the novel dye Exoria for the labeling of mesenchymal stromal cell-extracellular vesicle preparations. *Cytotherapy*. doi:10.1016/j.jcyt.2022.02.003
